# Clinical response to the canakinumab in crohn’s disease related artrhritis

**DOI:** 10.1186/1546-0096-12-S1-P346

**Published:** 2014-09-17

**Authors:** Zübeyde Gündüz, Ruhan Düşünsel, Duran Aslan, Betül Sözeri, Ayşenur Paç Kısaarslan

**Affiliations:** 1 Pediatric Rheumatology, Erciyes University faculty of medicine, Kayseri, Turkey; 2 Pediatric Gastroenterology, Erciyes University faculty of medicine, Kayseri, Turkey

## Introduction

Crohn’s disease (CD) is an inflammatory disorder of the gastrointestinal (GI) tract that is both chronic and relapsing. In addition to affecting the GI tract, CD has several extra-intestinal manifestations. Arthritis is a common, occurring in approximately 30% of CD patients. Here we report a patient with CD who had treatment resistant artrthritis.

## Results

A 4 years old girl was admitted because of right hip pain. When she was 1 year old was diagnosed with Crohn's disease and taken sulfasalazine and corticosteroid. She had septic arthritis in her right hip one year ago. On admission, we have found pain and limitation in right hip. Also she was growth retardation. In her laboratory findings, acute phase reactants were elevated (white blood cells :20 500 /mm³, Thrombocyte : 596 000/ mm³, ESH:120 mm/h, CRP 50,2 mg/L). She had also anemia (Hemoglobine : 8 gr/dl). We found ANA and HLA B27 were negative. We detected arthritis in right hip joint and bilateral sacroiliac joints in her MRI. Glucocorticoids and methotrexate (MTX) was started effectively; however, the patient did not reach complete remission. Therefore etanercept was added her therapy. We found homozygote MEFV mutation (M694V/M694V) and cholchine was added in her therapy.

After one year, a severe arthritis flare occurred, with an aggressive polyarticular course. In consideration of the lack of control obtained through the etanercept administration. We then decided to switch from etanercept to infliximab), which was administered at 7 dose. Despite this therapy, symptoms and laboratory findings did not regress. We started canakinumab (2mg/kg/month) therapy. Her arthritis was recovery on canacinumab in 3 months.

## Conclusion

Interleukin-1 (IL-1) is a highly active pro-inflammatory cytokine that lowers pain thresholds and damages tissues. Monotherapy blocking IL-1 activity in autoinflammatory syndromes results in a rapid and sustained reduction in disease severity, including reversal of inflammation-mediated loss of sight, hearing and organ function.

The pathogenesis of CD may be mediated by IL-1, and canakinumab may be useful when this disorder is unresponsive to more conventional treatments.

## Disclosure of interest

None declared

